# Propranolol in the preoperative treatment of Kasabach–Merritt syndrome: a case report

**DOI:** 10.1186/s13256-017-1475-0

**Published:** 2017-10-27

**Authors:** Saša V. Radović, Marija Kolinović, Darja Ljubić

**Affiliations:** 1 Pediatric Surgery Clinic, Institute for Childhood Diseases, Clinical Centre of Montenegro, Kruševac nn, 81000 Podgorica, Montenegro; 2 Pediatric Clinic, Institute for Childhood Diseases, Clinical Centre of Montenegro, Podgorica, Montenegro

**Keywords:** Kasabach–Merritt syndrome, Propranolol, Hemangioma, Infant, Platelets

## Abstract

**Background:**

Kasabach–Merritt syndrome represents the association of hemangioma with thrombocytopenia and consumptive coagulopathy. We present a case of Kasabach–Merritt syndrome treatment with orally administered propranolol.

**Case presentation:**

A 4.5-month-old caucasian female infant with congenital giant hemangioma in the posterior region of her neck presented to our Institute for Childhood Diseases where she underwent clinical, laboratory, and radiological investigations. A low blood platelet count indicated the use of corticosteroids and blood components as first-line therapy. The lack of therapeutic response induced the introduction of orally administered propranolol as additive therapy. A 3-week treatment led to a reduction in the size of hemangioma and a rise in platelet count which enabled surgical treatment and definite healing.

**Conclusion:**

Orally administered propranolol as monotherapy or in combination with other therapeutic modalities may play a key role in the treatment of Kasabach–Merritt syndrome.

## Background

Thrombocytopenia associated with giant hemangiomas was first described in 1940 by Kasabach and Merritt [1]. Since then, around 200 cases have been reported in the literature. This rare disorder may be present at birth or may appear later in infancy as the vascular malformation grows. More than 80% of cases manifest within the first year of life [2]. Estimated mortality ranges from 10 to 37%. Mortality and morbidity are usually associated with internal organ involvement, hemorrhage related to aggressive invasion, profound thrombocytopenia and disseminated intravascular coagulation (DIC), severe infections, and iatrogenic complications (http://www.emedicine.medscape.com/article/956136-overview). Kasabach–Merritt syndrome (KMS) shows wide variation in its response to different treatment modalities. Therefore, optimal therapy for this rare syndrome has not yet been established. Guidance for clinicians encountering patients with KMS is limited and because of the life-threatening and heterogeneous presentation of KMS, randomized controlled trials of these therapies, to yield evidence-based management protocols, are difficult to perform. Management strategies include use of corticosteroids, interferon, chemotherapy, propranolol, embolization, laser therapy, radiation and/or surgery. As the primary mechanism of platelet destruction is intravascular coagulation within the tumor, therapy should be aimed at controlling the coagulopathy by reducing the size of the tumor.

We report the successful treatment of a patient with KMS with propranolol, prednisolone, and surgical intervention, aiming to emphasize the importance and potential efficacy of propranolol in the treatment of KMS but also the challenges clinicians face in managing this rare and life-threatening syndrome.

## Case presentation

A 4.5-month-old caucasian female infant was presented in April 2016 to the Department of Hematology of the Institute for Childhood Diseases with an extremely low platelet count. The finding was a component of the evaluation prior to surgical treatment of the tumor on her neck, which seemed to be a congenital hemangioma.

She was the third child of non-consanguineous parents*,* from third healthy pregnancy, born at term by cesarean section: birth weight (BW) 4260 gr, birth length (BL) 57 cm, head circumference (HC) 37 cm, and Appearance, Pulse, Grimace, Activity and Respiration (APGAR) score 9/9. She was fed on adapted milk formula from the beginning. Her family history is unremarkable.

**Fig. 1:**
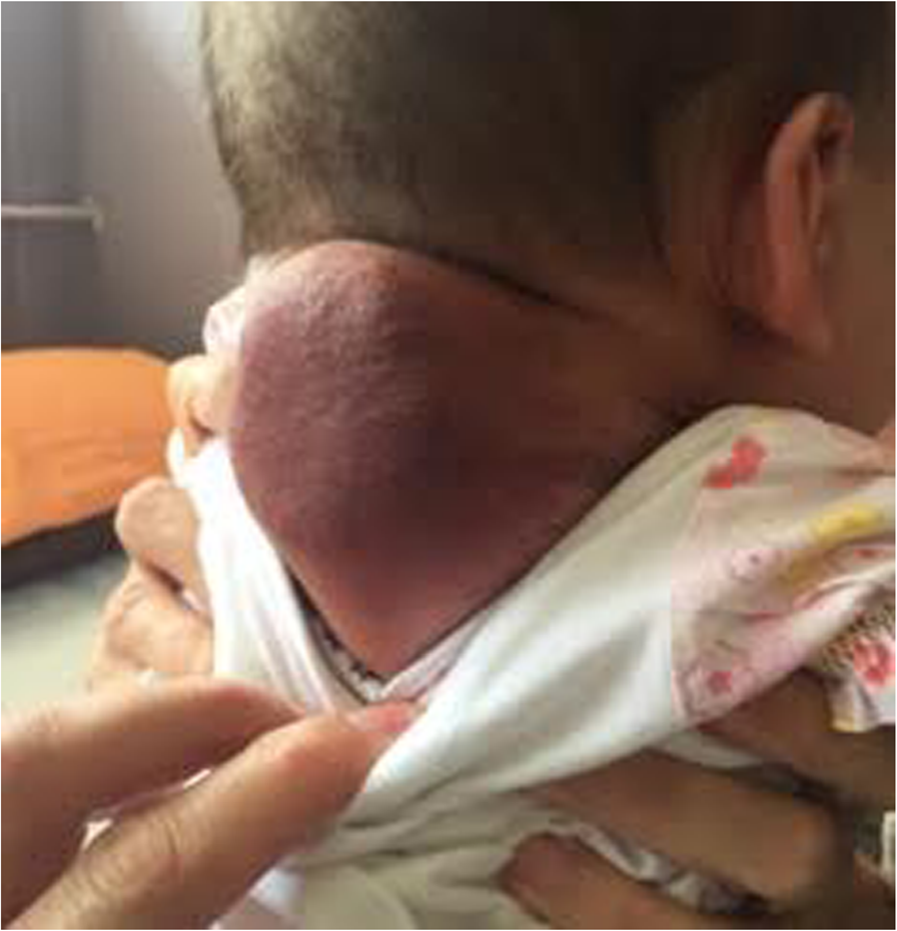
Soft tissue tumor of the posterior region of the neck in a 4.5-month-old baby girl

In the first hours after birth, a tissue mass in the cervical thoracic part of her vertebral column, which looked like a hematoma, measuring 1.5 × 1.5 cm was detected. In the further course of treatment the mass was defined as a tumor, 2 × 2 cm in size, which seemed to be a hemangioma (Fig. 1).

The child had been taken to regular check-ups until the indication for operative treatment was established. Her blood parameters (leukocyte formula and platelets) were within the normal range prior to preoperative evaluation.

On admission, the child was conscious, body mass (BM) 5900 gr, with adequate spontaneous and provoked activity, afebrile with a body temperature (BT) of 36.7 °C, and with normal respiratory and heart rate; she was acyanotic, anicteric, and had normal osteomuscular status. Her vital signs were in the normal ranges. In her neck, in suboccipital region and medially, existed a tumor of hemangioma type, size 6 × 6 cm, of firm consistency. The skin above the tumor was cyanotic, with petechiae, without signs of devitalization. Other physical findings were normal.

The values of some laboratory parameters were: white blood cells (WBC) 9.21 × 10^9^/l; red blood cells (RBC) 3.06 × 10^12^/l; hemoglobin (Hgb) 87 g/l; platelets 7 × 10^9^/l; partial thromboplastin time (PTT) 12.6 seconds; international normalized ratio (INR) 0.98 seconds; activated partial thromboplastin time (APTT) 41.6 seconds; fibrinogen 1.0 g/l; and D-dimer 26.9 mg/ml.

Doppler ultrasound of the soft tissue of the posterior aspect of her neck revealed an extensive, soft tissue heterogeneous mass, predominantly hyperechogenic, with areas of calcification and enhanced color Doppler (CD) signal, which may correspond to a hemangioma.

**Fig. 2:**
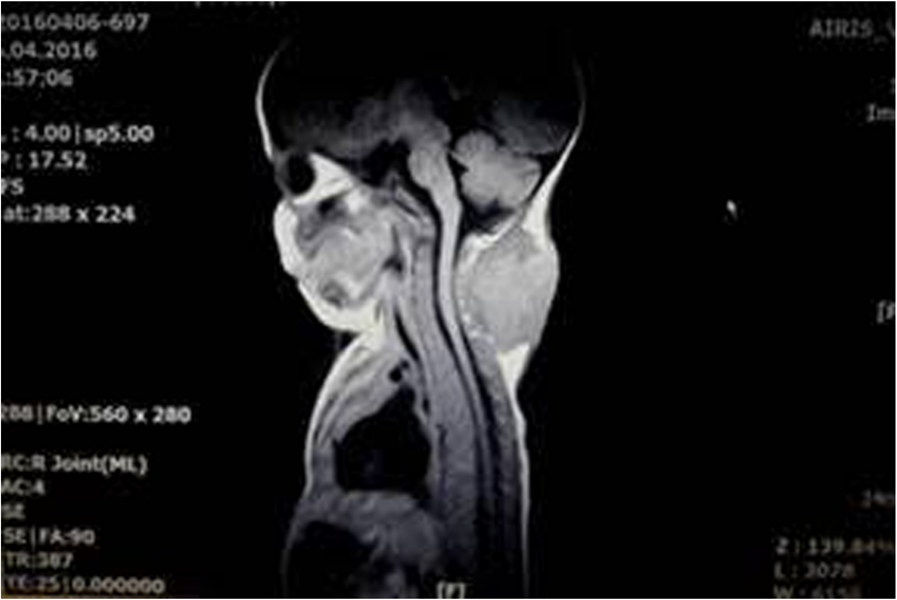
Magnetic resonance imaging of soft tissue mass in the neck – T1-weighted sequence

**Fig. 3:**
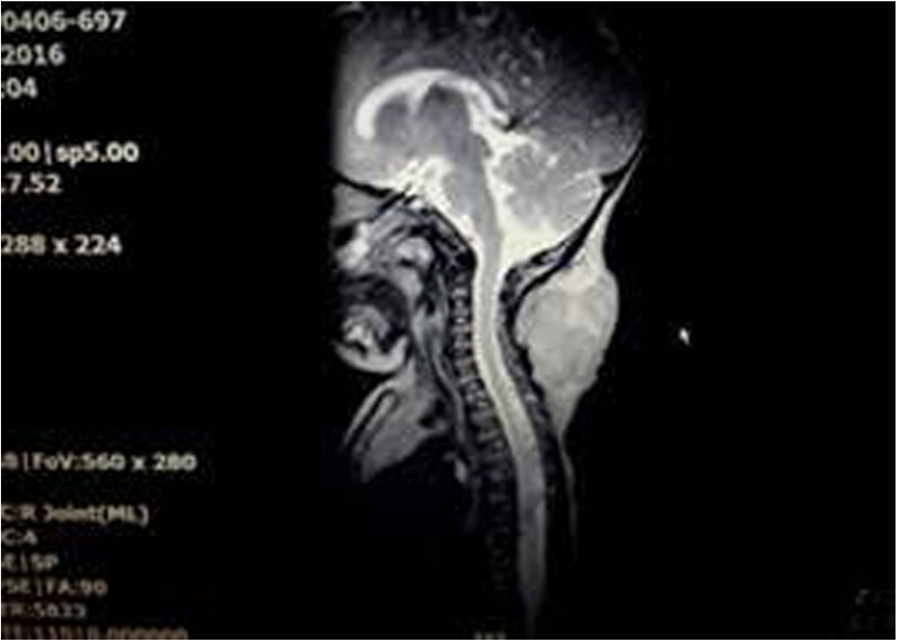
Magnetic resonance imaging of soft tissue mass in the neck – the contrast enhancement on T2-weighted sequence

A magnetic resonance imaging (MRI) examination revealed cutis and subcutaneous tissue of the posterior aspect of her neck, and a soft tissue, irregular, lobular mass, size 58 × 53 × 35 mm (craniocaudal × laterolateral × anteroposterior; Cc × LL × AP), which was completely hyperintense on T2, with single isointense to hypointense areas and isointense signal on T1, compared to surrounding structures. It was a completely nonhomogeneous structure, clearly bounded, not propagating toward the spinal canal with intensive inhomogeneous contrast enhancement. In conclusion, on MRI the mass had characteristics of benign soft tissue mass (Figs. 2 and 3).

As a result of our patient’s low platelet count and laboratory signs of consumptive coagulopathy, substitution with fresh frozen plasma (FFP) 15 ml/kg, along with intravenously administered corticosteroid (methylprednisolone) 2 mg/kg, were carried out daily during 7 days. The laboratory parameters improved: platelets 30 × 10^9^/l; fibrinogen 1.5 g/l; and D-dimer 25 mg/ml. The child was discharged home in good general condition. Her parents were advised to take her for a check-up at Hematology out-patient clinic in 7 days with complete blood count (CBC), fibrinogen, and D-dimer. The re-planning of surgical management was also indicated.

Six days after discharge, the child was readmitted. Her parents noticed enlargement of the tumor in her neck and laboratory tests revealed a low platelet count. Compared with previous clinical examination, the tumor was larger and was 8 × 8 cm in size. The values of some laboratory parameters were: WBC 9.5 × 10^9^/l; RBC 3.2 × 10^12^/l; Hgb 90 g/l; platelets 9 × 10^9^/l; PTT 13.5 seconds; INR 1.05 seconds; APTT 36.4 seconds; fibrinogen 2.1 g/l; and D-dimer 12.7 mg/ml. Urine culture was sterile, while *Staphylococcus aureus* was isolated from chemo-culture. A cardiologist was consulted. Her electrocardiogram and echocardiographic findings were normal.

Intravenously administered corticosteroid therapy (methylprednisolone) 2 mg/kg, FFP 15 mg/kg, and symptomatic therapy were administered. Ten days after admission, a rise in her body temperature occurred. Her inflammatory parameters such as C-reactive protein (CRP) 6.8 mg/l were monitored and the symptomatic treatment was continued. The child also received RBC transfusion because of low Hgb values. The values of some laboratory parameters, 7 days after discontinuation of therapy, were: WBC 8.5 × 10^9^/l; RBC 4.2 × 10^12^/l; Hgb 112 g/l; platelets 25 × 10^9^/l; PTT 13.5 seconds; INR 1.05 seconds; APTT 36.4 seconds; fibrinogen 2.4 g/l; and D-dimer 10.9 mg/ml.

**Fig. 4:**
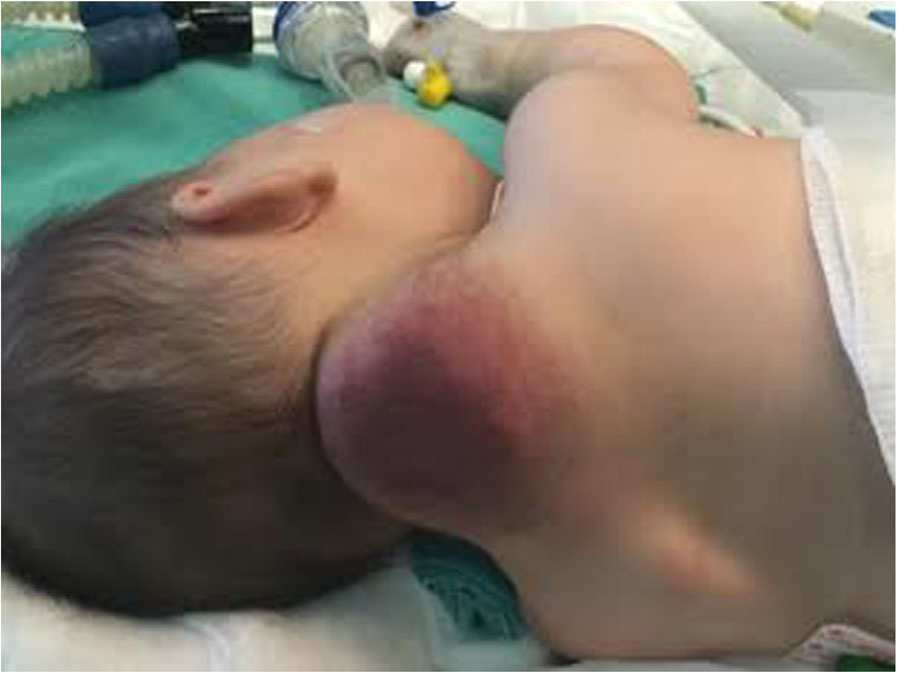
Preoperative view – the reduction in tumor size due to treatment with propranolol

After 14 days of the second hospitalization, in consultation with a pediatric surgeon, treatment with propranolol was initiated at a total daily dose of 0.5 mg/kg divided into three doses. Her blood pressure, heart rate, and blood glucose were regularly monitored. On the second day, the dose was increased to 1 mg/kg, then to 2 mg/kg and on the fourth day, to 3 mg/kg. The values of monitored parameters were in physiological ranges. The control platelet counts were continuously increasing (from 5 × 10^9^/l to 100 × 10^9^/l) within 2 weeks, along with an improvement of coagulation status and reduction of hemangioma size to 5 × 5 cm. After 30 days of hospitalization, she was transferred to Department of Pediatric Surgery for further treatment (Fig. 4).

**Fig. 5:**
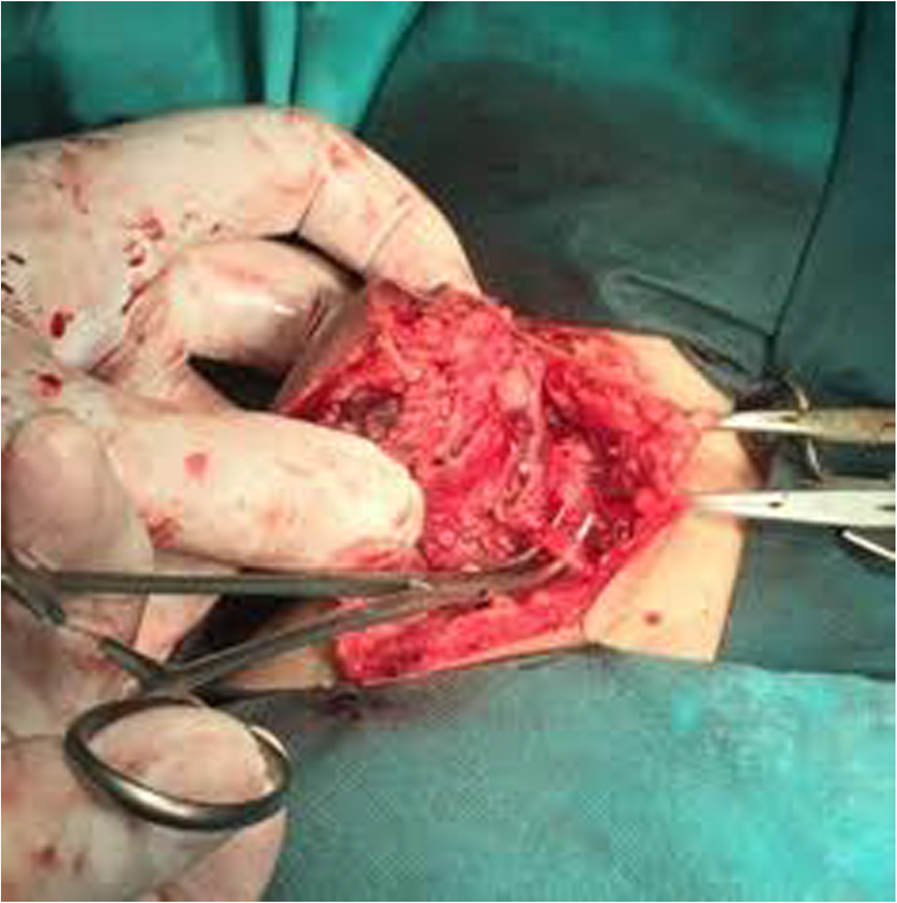
Intraoperative view – the nutrient artery of the tumor

**Fig. 6:**
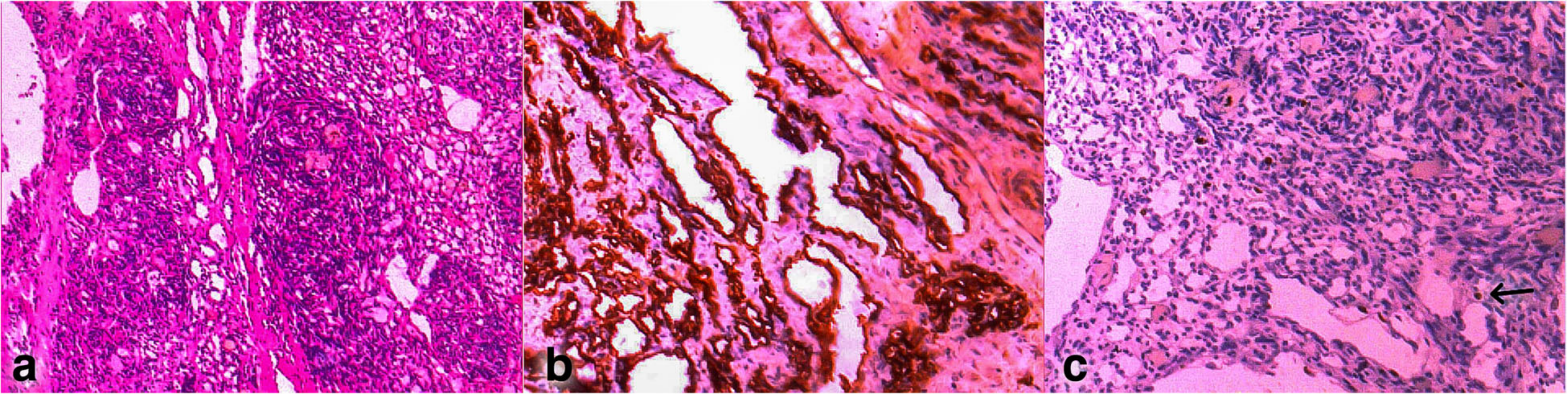
Histopathological findings. **a** Hematoxylin and eosin stain; **b** immunohistochemical analysis of tissue – CD34 positive; **c** immunohistochemical analysis of tissue – Ki67 labeling index (1%)

After 3 weeks of propranolol therapy, the child underwent the surgical extirpation of the tumor. The tumor tissue was approached through an elliptical incision in normal skin area. By careful preparation, it was separated from the normal subcutaneous tissue. Among the thrombosed vasculature, the nutrient blood vessel was identified (Fig. 5). The tumor was dissected down to the prevertebral fascia and removed. Hemostasis was achieved and a drain was placed in the tumor bed. Her early postoperative period was uneventful. The tumor was sent for histopathological (HP) examination which revealed well-circumscribed lobules of closely packed capillaries composed of plump endothelial cells and pericytes, separated by normal-appearing dermal stromal elements (hematoxylin and eosin stain; Fig. 6a). Immunohistochemical analysis of tissue was as follows: CD34 positive (Fig. 6b); Ki67 labeling index = 1% (Fig. 6c); and cells were positive for vimentin and negative for epithelial membrane antigen (EMA) and pancytokeratin (CK). Based on clinical and HP features, a definite diagnosis of hemangioma capillare cutis was made.

Dual antibiotic therapy (ceftriaxone, amikacin) was given postoperatively. The wound was cleaned and the dressing changed on a regular basis. The wound was clean and dry, and was healing by first intention. Sutures were removed on 7th postoperative day. The laboratory tests on the 3rd postoperative day showed normalization of CBC parameters and coagulation status.

**Fig. 7:**
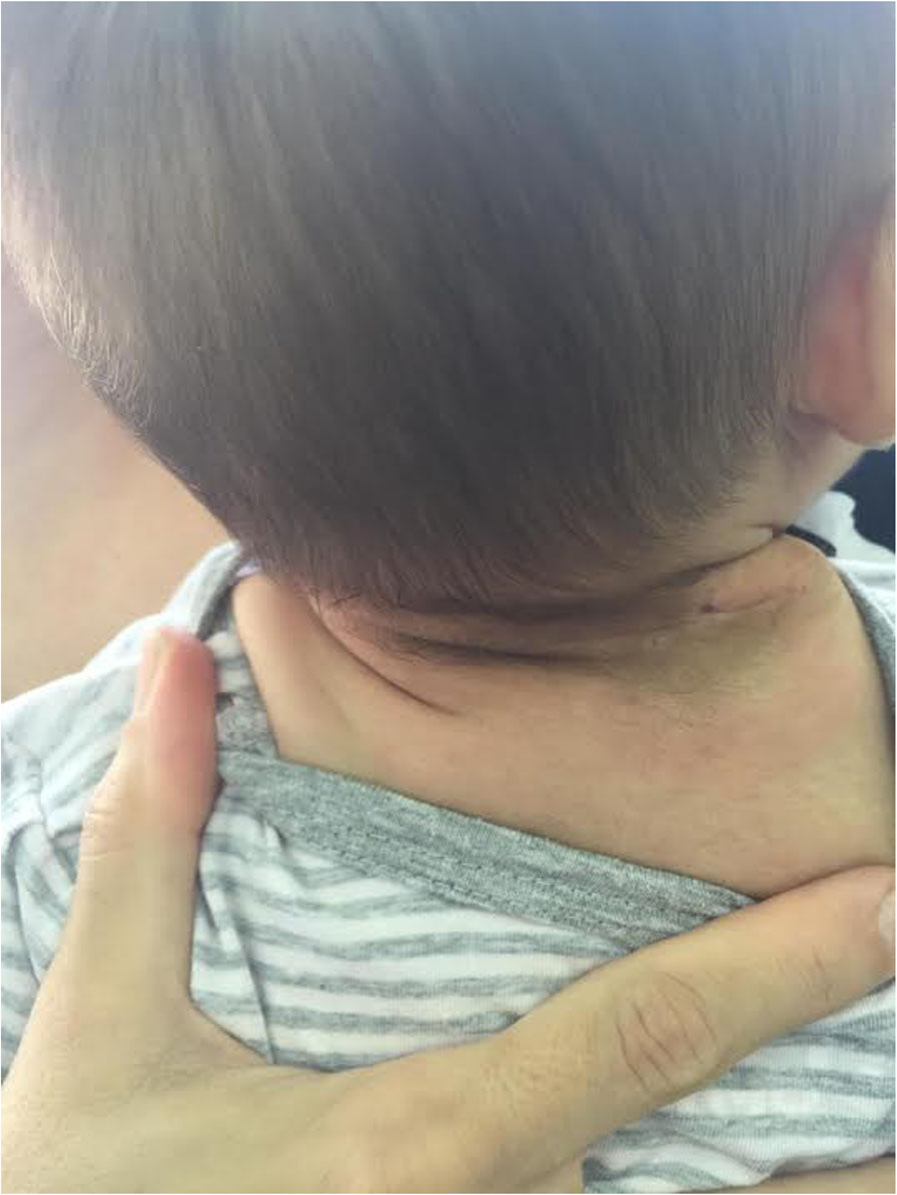
Postoperative view 4 weeks after surgical treatment

She was discharged in good general condition on the 7th postoperative day with follow-up appointments with hematologist every 7 days for the first month, and then once a month for up to 12 months, along with the results of laboratory tests, platelet counts, and coagulation status parameters, which were within physiological ranges (Fig. 7).

## Discussion

Basic diagnostic criteria for KMS are the presence of giant hemangioma and low platelet count. KMS should be always suspected in children with unexplained thrombocytopenia and coagulation disorders. Clinical manifestations may appear at birth or during the first 12 to 18 months of life. Routine blood tests show varying degrees of low Hgb concentration, low plasma level of fibrinogen, and prolonged prothrombin time. Ultrasonography (B-mode), computed tomography (CT), and MRI reveal the size, appearance, and layers of hemangioma, as well as its relationships with peripheral blood vessels, and distinguish hemangioma from vascular malformations. In cases of unclear clinical presentation, along with etiologically unexplained thrombocytopenia, scintigraphy with chromium-51/indium-111 oxine-labeled platelets or iodine-131-labeled fibrinogen, increases sensitivity in making a diagnosis. KMS is associated with kaposiform hemangioendothelioma in over 90% of cases and uncommonly with infantile (cavernous or capillary) or congenital hemangioma [3].

The management of KMS is very challenging because of its rarity and unstandardized therapeutic strategies. Systemic corticosteroids are considered to be first-line therapy for this syndrome [4]. If the tumor responds to steroids, the dose should be reduced slowly in order to prevent recurrence. The mechanisms of prednisolone in controlling thrombocytopenia, coagulopathy, and stabilization of hemangioma are still unclear, but it appears that it increases the number of platelets and vasoconstriction, inhibits fibrinolysis, and disrupts angiogenesis [5]. In cases resistant to systemic corticosteroids, multiple treatment options can be used in a stepwise manner, including antiproliferative and antiangiogenic agents; interferon-alpha and vincristine are strong inhibitors of angiogenesis. Radiotherapy is another option which may induce embolization within the hemangioma. These options are usually recommended as second-line treatments [6]. Surgical intervention may be the therapy of first choice if it will not destroy the patient’s appearance or function. Complete removal of the tumor can cure KMS [7].

Since the first report in 2008, propranolol has showed many advantages in the treatment of infantile hemangioma [8]. The use of propranolol is new and encouraging, but it requires careful monitoring for complications. The most common reported adverse effects of propranolol are hypotension, bradycardia, hypoglycemia, and bronchospasm. In infantile hemangiomas, the possible mechanism of action of propranolol is vasoconstriction, decreased expression of vascular endothelial growth factor (VEGF) and basic fibroblast growth factor (bFGF) genes through the down-regulation of the RAF (serine/threonine-protein kinase)-MAPK (mitogen-activated protein kinase) pathway (which explains the regression of the hemangioma), and the triggering of apoptosis of capillary endothelial cells [9].

There are still controversies regarding the effect of propranolol on KMS. There are only a few literature reviews on good [10, 11] and poor [12, 13] responses to propranolol, when used in addition to a steroid or vincristine treatment for KMS.

Choeyprasert and associates suggested that propranolol as monotherapy may be useful in mild KMS (initially small size of the primary tumor, 5 × 5 cm; and the mild severity of coagulopathy, platelets 55 × 10^9^/L), at a dose of 2 mg/kg per day, divided into three doses [11]. This is also supported by the study of Chiu and colleagues on variable responses to propranolol monotherapy of three patients with KMS: two patients did not respond to propranolol, while one patient, with tumor 5 cm in size, responded completely to treatment [12]. It was concluded that primary vascular tumors less than 5 to 6 cm in size might respond better than larger ones to propranolol treatment, and that the severity of coagulopathy, including degree of thrombocytopenia, plasma D-dimer, and fibrinogen levels, might also determine responsiveness to treatment. In the literature, most patients with KMS who did not respond or only had a partial response to propranolol had severe thrombocytopenia and significantly elevated D-dimer levels [11].

The size of hemangioma in our case is in line with the conclusions found in the literature and referred to above, but that is not the case with low platelet count. Despite the severity of thrombocytopenia, our patient had an excellent response to the treatment which was not expected according to the above mentioned data from the literature. Therefore, our case report suggests that propranolol therapy should be taken into consideration regardless of significant thrombocytopenia.

Dhandore and associates reported inefficiency of steroids and propranolol in the treatment of KMS: a 24-hour-old full term male child with giant hemangioma over right thigh since birth along with thrombocytopenia and coagulopathy. Vincristine and interferon were not taken into consideration due to the child’s clinical condition. An emergency surgical excision was performed to prevent life-threatening multiorgan hemorrhage [14].

## Conclusions

This case report emphasizes the importance and potential efficacy of propranolol in the treatment of KMS. An excellent treatment response enabled surgical intervention and definite management of tumor, along with normalization of platelet count and coagulation status. Considering the fact that the treatment of KMS is still empirical, it is extremely important to publish individual experiences in management of this rare and life-threatening syndrome.

## Abbreviations

AP: Anteroposterior
APGAR: Appearance, Pulse, Grimace, Activity, and Respiration
APTT: Activated partial thromboplastin time
bFGF: Basic fibroblast growth factor
BL: Birth length
BM: Body mass
BT: Body temperature
BW: Birth weight
CBC: Complete blood count
CC: Craniocaudal
CD: Color Doppler
CK: Pancytokeratin
CRP: C-reactive protein
CT: Computed tomography
DIC: Disseminated intravascular coagulation
EMA: Epithelial membrane antigen
FFP: Fresh frozen plasma
HC: Head circumference
Hgb: Hemoglobin
HP: Histopathological
INR: International normalized ratio
KMS: Kasabach–Merritt syndrome
LL: Laterolateral
MAPK: Mitogen-activated protein kinase
MRI: Magnetic resonance imaging
PTT: Partial thromboplastin time
RBC: Red blood cells
VEGF: Vascular endothelial growth factor
WBC: White blood cells

## References

[1] Kasabach NH, Merritt KK. Capillary hemangioma with extensive purpura. Am J Dis Child. 1940;59:1063–70. doi:10.1001/archpedi.1940.01990160135009.

[2] Martins AG. Hemangioma and thrombocytopenia. J Pediatr Surg. 1970;5:641–8. 10.1016/s0022-3468(70)80010-0 5533877

[3] Wang P, Zhou W, Tao L, Zhao N, Chen XW. Clinical analysis of Kasabach-Merritt syndrome in 17 neonates. BMC Pediatr. 2014;14:146. doi:10.1186/1471-2431-14-146. PMC4088914 24920221

[4] Drucker AM, Pope E, Mahant S, Weinstein M. Vincristine and corticosteroids as first-line treatment of Kasabach-Merritt syndrome in kaposiform hemangioendothelioma. J Cutan Med Surg. 2009;13:155–9. 10.2310/7750.2008.08006 19426625

[5] Tarek A, Rabiul H, Achira B, Tahmina JC. A Case Report on Kasabach Merrit Syndrome. Bangladesh J Child Health. 2012;36(2):105–7. doi:10.3329/bjch.v36i2.13091.

[6] Rodriguez V, Lee A, Witman PM, Anderson PA. Kasabach merritt phenomenon: Case series and retrospective review of the Mayo Clinic experience. J Pediatr Hematol Oncol. 2009;31:522–6. doi:10.1097/MPH.0b013e3181a71830. 19564750

[7] Velin P, Dupont D, Golkar A, Valla JS. Neonatal Kasabach-Merritt syndrome healed by complete surgical excision of the angioma. Arch Pediatr. 1998;5:295–7. doi:10.1016/S0929-693X(97)89373-6. 10327999

[8] Sans V, de la Roque ED, Berge J, Grenier N, Boralevi F, *et al.* Propranolol for severe infantile hemangiomas: follow-up report. Pediatrics. 2009;124:423–31. doi:10.1542/peds.2008-3458. 19706583

[9] Guldbakke KK, Rørdam OM, Huldt-Nystrøm T, Hanssen HK, Høivik F. Propanolol used in treatment of infantile hemangioma. Tidsskr Nor Laegeforen. 2010;130:1822–4. doi:10.4045/tidsskr.09.1435. 20882080

[10] Hermans DJJ, van Beynum IM, van der Vijver RJ, Kool LJS, de Blaauw I, *et al.* Kaposiform hemangioendothelioma with Kasabach-Merritt syndrome: a new indication for propranolol treatment. J Pediatr Hematol Oncol. 2011;33(4):171–3. doi:10.1097/MPH.0b013e3182152e4e. 21516018

[11] Choeyprasert W, Natesirinilkul R, Charoenkwan P. Successful treatment of mild pediatric Kasabach-Merritt phenomenon with propranolol monotherapy. Case Rep Hematol. 2014;22:364693. doi:10.1155/2014/364693. ID 364693. PMC4054805 24963423

[12] Chiu YE, Drolet BA, Blei F, *et al.* Variable response to propranolol treatment of kaposiform hemangioendothelioma, tufted angioma, and Kasabach-Merritt phenomenon. Pediatr Blood Cancer. 2012;59(5):934–8. doi:10.1002/pbc.24103. PMC3528889 22648868

[13] Hall GW. Kasabach-Merritt syndrome: pathogenesis and management. Br J Haematol. 2001;112(4):851–62. doi:10.1046/j.1365-2141.2001.02453.x. 11298580

[14] Dhandore PD, Hombalkar NN, Pancholi CK. Kasabach Merritt Syndrome: Is there a Role of Surgery? J Clin Neonatol. 2016;5(1):58–60. doi:10.4103/2249-4847.173280.

